# Industrial Investment Funds, Government R&D Subsidies, and Technological Innovation: Evidence From Chinese Companies

**DOI:** 10.3389/fpsyg.2022.890208

**Published:** 2022-06-14

**Authors:** Yuan-ming Ren

**Affiliations:** Management Committee of Rongchang Campus, Southwest University, Chongqing, China

**Keywords:** industrial policy, government subsidies, R&D investment, technological innovation, sustainability

## Abstract

Industrial investment funds are a new financing innovation mode that can build an effective financing channel for enterprises.Based on the panel data of Chinese Listed Companies in 2008–2017, this manuscript constructed a static panel model between industrial investment funds, government R&D subsidies, and technological innovation to empirically analyze the effects of industrial investment fund involvement and government R&D subsidies on companies’ technological innovation. The research shows that industrial investment fund involvement can increase the company’s R&D investment by providing financial funds for the company, which can effectively solve the company’s lack of funds in the process of technological innovation and guarantee the smooth running of the company’s innovation activities. Secondly, government R&D subsidies can alleviate the pressure of R&D investment to a certain extent, which is conducive to promote a higher level of technological innovation in the company. Thirdly, for companies with industrial investment fund involvement, government R&D subsidies are conducive to promote technological innovation. In contrast, for companies without industrial investment fund involvement, government R&D subsidies have no significant impact on technological innovation to a certain extent or even have a “crowding out effect.”

## Introduction

Technological innovation is the key to enhance a company’s market competitiveness and ensure its sustainable operation. Economic globalization and fierce market competition drive companies to attach importance to R&D innovation, but lack of capital is an important obstacle that affects such companies’ technological innovation. At present, although companies can obtain funds for research and innovation by going public, the capacity of the capital market is very limited, and direct financing methods do not perform the function of capital financing. Therefore, many small- and medium-sized companies cannot seek funding in this way. The industrial investment fund^1^ system is set up to effectively solve the financing problems, giving the company corresponding financial support by attracting a large amount of idle social capital. It may also effectively supervise the projects or companies it invests in and participates in its management. Industrial investment funds can provide new financing channels for companies and solve the problem of insufficient funds for R&D, which will affect the level of technological innovation of companies.

As such, this manuscript considered the following important questions regarding this topic: How do industrial investment funds influence companies’ technological innovation? What are the effects of industrial investment funds on a company’s technological innovation? How can the advantages of industrial investment funds be made full use of to improve the technological innovation level of a company? Answering the above questions will help policymakers deepen their understanding of industrial investment funds, effectively improve the level of technological innovation of Chinese companies, and then use the unique advantages of industrial investment funds to promote the development of Chinese industries.

## Theoretical Analysis and Research Hypothesis

### Industrial Investment Funds and Technological Innovation

Industrial investment funds can widen financing channels, alleviate financing constraints, improve financing efficiency, and increase the capital for technological innovation, thus contributing to a firm’s technological innovation.Arizala et al. (2013); Gehringer (2013), and Wallsten (2000) Economic globalization and fierce market competition drive companies to focus on development and innovation to update their products through technological innovation and thus meet the customer demands. However, the capital shortage is also an important aspect that affects companies’ technological innovation. Existing experience shows that long-term capital investment is required to enhance a company’s technological innovation capability. Many companies’ R&D projects are terminated due to insufficient follow-up funds, which has been a universal problem in company’s R&D (Mueller, 1966; Gang and Weidong, 2017). Before the advent of industrial investment funds, investment tools in the market were mainly financial assets, such as stocks, bonds, foreign exchange, and futures. For innovation, it is important for companies to be able to transform savings into financial capital, but they cannot do it directly. Companies can use stocks, bonds, foreign exchange, futures, and other financial assets to meet daily financial support but cannot provide stable support for technological innovation. In the long run, indirect financing has occupied a dominant position in China’s financing system, and indirect financing is likely to trigger information asymmetry between the two sides of the transaction. Financial frictions, such as information asymmetry, often cause companies to face higher external financing costs when investment opportunities arise, resulting in higher external financing costs than internal funds, exposing companies to different financing constraints. Commercial banks have difficulty in finding target customers who meet the credit conditions, and there is uncertainty about whether the company can successfully obtain capital financing. The preferred financing channel for many companies is the commercial bank credit. Still, indirect financing has prominent interests and policy preference features, making it difficult for some industries, especially high-tech industries, strategic emerging industries, and infrastructure construction industries, to meet their own capital needs with indirect financing (Grant, 1991; Moffat, 2014). Preferential financing theory suggests that another option for corporate financing is financial market financing, which is direct financing.

However, due to the limited capacity of the current market in China, many companies are still not eligible to obtain financing through the financial market. Even if they are, most of the funds revert back to the financial market. For instance, many companies allocate the funds that they raise to the financial market, which is operated in the primary and secondary markets, and even allocate them to real estate and other fields to obtain additional returns (Orhangazi, 2008; Lapavitsas, 2011; Ju et al., 2013; Wang et al., 2017; Cai et al., 2019). Therefore, the direct financing method has not fulfilled the function of capital financing but has led to false prosperity in the financial market and a latent financial bubble. Thus, it is evident that the clogged financing channels of companies are the fundamental reasons for the lack of innovation motivation. In these scenarios, companies lack funds to expand production scale, update production equipment, and develop new products. Thus, the industrial investment fund system is set up to effectively crack the financing problems of a company.

Industrial investment funds give corresponding financial support to a company by attracting a large amount of idle social capital. Specifically, the industrial investment funds are invested in selected projects, and companies go through feasibility studies. The industrial investment fund effectively supervises the projects or companies invested during the dynamic operation of the investment funds. It participates in the management of the company at the right time, which affects the level of technological innovation of the company by changing the company’s financing structure. In addition, once the company has less liquidity, it can apply to the industrial investment fund for additional investment funds. When the company’s R&D investment is insufficient, it can obtain the required funds from the specific industrial investment funds as long as it is credible and meets the investment requirements. Thus, providing a new financing channel for the company and solving the problem of insufficient funds for R&D investment can place a company’s sustainable operation and technological innovation on a healthy development track. To this end, this manuscript proposes:

H1: The involvement in industrial investment funds positively correlates with a company’s technological innovation.

### Industrial Investment Funds, Government R&D Subsidies, and Technological Innovation

Many studies in academic circles point out that government incentives^2^ can improve credit availability and thus affect the R&D investment of companies (Guellec and Pottelsberghe, 2003; Xinglong et al., 2014; Chu et al., 2016). First, government R&D subsidies, as external financial support for the company, will have an incentive effect on the company’s R&D investment to a large extent. On the one hand, government R&D subsidies offset the company’s expenditures and reduce the actual costs. In contrast, government R&D subsidies improve the company’s enthusiasm. It plays the role of risk compensation, solves the problems, and enhances the company’s expected revenue while creating positive incentives for its R&D investment. Secondly, government R&D subsidies alleviate the potential risks of the company’s R&D activities (Ehie and Olibe, 2010). The company needs to concentrate on investing considerable capital resources for technological innovation. The R&D cost expenditure will directly affect the company’s financing structure, increasing the company’s operational pressure to a certain extent. Government R&D subsidies, to some degree, also alleviate the liquidity and operational risk arising from the company’s capital investment in R&D activities. By contrast, the company’s R&D investment faces great uncertainty, which means that the company needs to bear the risk of R&D failure.

Government R&D subsidies mean that the company has sufficient funds to support R&D activities, and from the input point of view, it improves the expected return of the company’s R&D innovation. At the same time, it also compensates for the loss caused by some companies’ R&D failure. It enhances the confidence of companies that are afraid to invest and thus increases their innovation investment. In addition, R&D results have a strong externality, so the spillover effect of R&D results can be easily imitated by other companies, leading to lower expected revenue. Finally, government R&D subsidies also serve as a positive signal for establishing cooperative links between government and enterprises. The company’s disclosure of government R&D subsidies will significantly increase the company’s social attention, which will significantly improve the company’s market evaluation. A company receiving government R&D subsidies will have a well-established market image, so it may attract more investors’ attention, allowing it to obtain more funds in the capital market. Therefore, to implement a government R&D subsidy policy, the company may obtain a government R&D subsidy by increasing R&D investment, thus indirectly enhancing the absorption force of its resources and improving the level of its R&D.

Simultaneously, there may also be a substitution relationship between government R&D subsidies and R&D innovation. Companies will strive to have good relations with the government to receive additional subsidies to meet the government policy and reform orientation. At the same time, they may use part of the government R&D subsidies for R&D innovation, thus generating a “crowding-out effect.” Specifically, first, the crowding-out effect of government R&D subsidies is caused mainly due to the increased demand for R&D factors. Government R&D subsidies are used for the increased price of the factors. For example, companies will invest more money in R&D and hire higher-end technology teams, which pushes up those companies’ costs, leading to a decrease in their willingness to conduct R&D and eventually making companies reduce their R&D investment.

Further, government R&D subsidies, as a component of government fiscal expenditure, will have a specific moderating effect on the supply and demand of the market economy (Teng et al., 2021; Hang et al., 2022). Since scientific research personnel (STI) personnel, equipment, and materials are inelastic in the short term, government R&D subsidies will, to a certain extent, intensify companies’ demand for R&D factors, which means that the price of market R&D factors (STI personnel, equipment, and materials, etc.) will rise to increase the R&D cost and lower the expected revenue, which will prompt the company to give up R&D and shift the capital to other higher-yielding investments. In particular, companies with no or fewer subsidies, whose expected benefit will be significantly lowered, will undoubtedly reduce their R&D investment, an obvious example of the “crowding out” effect of government R&D subsidies.

Secondly, government R&D subsidies help to foster a research atmosphere for companies (Shah et al., 2019a,b). However, the information asymmetry between the government and the company will cause such companies to face moral risks. For example, when a company’s industry has relatively low R&D costs, that company will use government R&D subsidies to replace its research investment to reduce expenditure and use government R&D subsidies as its other daily expenses to meet its liquidity needs, thus transferring government R&D subsidies to other purposes. Therefore, the company’s R&D investment is overly reliant on government R&D subsidies, which, on the one hand, reduces the company’s enthusiasm for independent innovation (Ajaz et al., 2020; Sarfraz et al., 2020). On the other hand, it may directly affect its R&D results, thus inhibiting its technological innovation level. At this time, government R&D subsidies will crowd out the current R&D innovation activities of the company and may even hinder the company’s technological innovation. For this reason, this manuscript proposes:

*H2: Government R*&*D subsidies positively correlate with a company’s technological innovation.*
*H3: Government R*&*D subsidies have a more significant impact on a company’s technological innovation with industrial investment fund involvement.*

## Model Setting

According to the previous analysis, industrial investment funds and government R&D subsidies may influence a company’s technological innovation. This manuscript first constructed a static panel model between industrial investment funds, government R&D subsidies, and technological innovation, which are denoted as model 1.


(1) $$\begin{array}{c} I\,N\,N\,O_{i\,t}=C_{1}+\alpha_{1}\,F\,U\,N\,D_{i\,t}+\alpha_{2}\,G\,R\,A\,N\,T_{i\,t}+\alpha_{3}\,G\,R\,O\,W_{i\,t}\\ +\alpha_{4}\,I\,N\,C\,O_{i\,t}+\alpha_{5}\,N\,E\,T_{i\,t}+\alpha_{6}\,D\,E\,B\,T_{i\,t}+\alpha_{7}\,I\,N\,T\,E_{i\,t}\\ +\alpha_{8}\,S\,I\,Z\,E_{i\,t}+\alpha_{9}\,L\,A\,B\,O\,R_{i\,t}+\alpha_{10}\,A\,G\,E_{i\,t}+v_{i}+u_{t}+\varepsilon_{i\,t} \end{array}$$

In Eq. (1), C1 is a constant term; INNO is the firm’s technological innovation; and FUND and GRANT are industrial fund involvement and government R&D subsidy. Meanwhile, to minimize endogeneity problems arising from omissions, this manuscript needed to control important variables that affected the level of technological innovation of the company. Without loss of generality, the control variables introduced in this manuscript were growth (GROW), the growth rate of operating income (INCO), net cash flow in operating activities (NET), total debt (DEBT), interest coverage ratio (INTE), asset size (SIZE), number of research staff (LABOR), and firm age (AGE). Where, α denotes the estimated coefficients of the explanatory variables; i is the individual firm and *t* is the year; vi and ut denote individual effects and time effects, respectively; and ε is the random error term.

To ensure the robustness of the conclusions, this manuscript introduced the first-order lagged variable of technological innovation as an explanatory variable based on equation (1).


(2) $$\begin{array}{c} I\,N\,N\,O_{i\,t}=C_{2}+\beta_{1}\,I\,N\,N\,O_{i\,t-1}+\beta_{2}\,F\,U\,N\,D_{i\,t}+\beta_{3}\,G\,R\,A\,N\,T_{i\,t}\\ +\beta_{4}\,G\,R\,O\,W_{i\,t}+\beta_{5}\,I\,N\,C\,O_{i\,t}+\beta_{6}\,N\,E\,T_{i\,t}+\beta_{7}\,D\,E\,B\,T_{i\,t}\\ +\beta_{8}\,I\,N\,T\,E_{i\,t}+\beta_{9}\,S\,I\,Z\,E_{i\,t}+\beta_{10}\,L\,A\,B\,O\,R_{i\,t}+\beta_{11}\,A\,G\,E_{i\,t}\\ +v_{i}+u_{t}+\varepsilon_{i\,t} \end{array}$$

In Eq. (2), C2 is a constant term; INNOit-1 denotes the first-order lagged variable of the firm’s technological innovation level, and the rest of the variables have the same meaning as equation (1). To address the endogeneity of the model, this manuscript adopted the generalized method of moments (GMM) of dynamic panels to estimate the model.

To examine whether government R&D subsidies of companies with and without industrial investment fund involvement significantly affect their technological innovation, we used the following equation:


(3) $$\begin{array}{c} I\,N\,N\,O_{i\,t}=C_{3}+\theta_{1}\,F\,U\,N\,D_{i\,t}\times G\,R\,A\,N\,T_{i\,t}+\theta_{2}\,G\,R\,O\,W_{i\,t}\\ +\theta_{3}\,I\,N\,C\,O_{i\,t}+\theta_{4}\,N\,E\,T_{i\,t}+\theta_{5}\,D\,E\,B\,T_{i\,t}+\theta_{6}\,I\,N\,T\,E_{i\,t}\\ +\theta_{7}\,S\,I\,Z\,E_{i\,t}+\theta_{8}\,L\,A\,B\,O\,R_{i\,t}+\theta_{9}\,A\,G\,E_{i\,t}+v_{i}+u_{t}+\varepsilon_{i\,t} \end{array}$$

(4) $$\begin{array}{c} I\,N\,N\,O_{i\,t}=C_{4}+\omega_{1}\,I\,N\,N\,O_{i\,t-1}+\omega_{2}\,F\,U\,N\,D_{i\,t}\times G\,R\,A\,N\,T_{i\,t}\\ +\omega_{3}\,G\,R\,O\,W_{i\,t}+\omega_{4}\,I\,N\,C\,O_{i\,t}+\omega_{5}\,N\,E\,T_{i\,t}+\omega_{6}\\ D\,E\,B\,T_{i\,t}+\omega_{7}\,I\,N\,T\,E_{i\,t}+\omega_{8}\,S\,I\,Z\,E_{i\,t}+\omega_{9}\,L\,A\,B\,O\,R_{i\,t}\\ +\omega_{10}\,A\,G\,E_{i\,t}+v_{i}+u_{t}+\varepsilon_{i\,t} \end{array}$$

where *FUND*_*it*_×*GRANT*_*it*_ indicates the impact of the interaction between industrial investment funds and government R&D subsidies on technological innovation, with FUND = 0 when the firm has no industrial investment fund involvement and FUND = 1 when the firm has one. The regression coefficient is used to determine whether the interaction between industrial investment funds and government R&D subsidies has a “crowding-in effect” or a “crowding-out effect” on technological innovation.

### Description of the Selection of Indicators

In this manuscript, the explanatory variables, core explanatory variables, and indicators of variables affecting firm technological innovation were screened using the existing literature.

#### Explanatory Variables

##### Technological Innovation (INNO)

R&D expenditure has traditionally been used as an indicator of technological innovation, so capability does not fully reflect the technological R&D and technological innovation capability of a company. Intangible assets are more reflective of a company’s technological innovation capability as compared to R&D expenditure. Therefore, using intangible assets to measure technological innovation is more reasonable.

#### Core Explanatory Variables

##### Industrial Investment Funds (FUND)

This manuscript is based on the companies listed in the initial public offering of China’s A-share main board market, small- and medium-sized board market, and Growth Enterprise market after 2008, excluding financial companies, non-normal type listed companies, and companies with missing data. Finally, 627 initial public offerings listed companies were obtained as the research sample. The data of industrial investment funds in this manuscript were obtained by consulting the prospectus of an initial public offering of listed companies on the Shanghai and Shenzhen stock exchanges, China Venture Capital Yearbook, financial sector, Juchao information, etc. Subsequently, the number of companies with industrial investment fund involvement in this manuscript is 76.

##### R&D Subsidies (GRANT)

Research and development subsidies alleviate the liquidity risk and operational risk arising from the capital investment of the company’s R&D activities to a certain extent. This manuscript had drawn lessons from Shanshan (2009) and Wang (2013) by selecting government R&D subsidies of listed companies to portray this indicator.

#### Control Variables

##### Growth (GROW)

Stable growth of net profit is the premise and guarantee of the company’s sustainable development; to grow the accuracy and rigor of the conclusions of this manuscript, the annual net profit growth rate of listed companies was selected as the main index to portray the growth of the company.

##### Increase Rate of Main Business Revenue (INCO)

The increased rate of main business revenue is the ratio of the increase of the company’s operating income in a certain period to the total operating income in the previous period. Overall, the higher the increase rate of main business revenue, the stronger the company’s growth and development ability, and therefore, the more investment in technology research and development there is likely to be.

##### Net Cash Flow From Operating Activities (NET)

Net cash flow from operating activities directly reflects the company’s operating results and is a prerequisite and guarantee for the sustainable operation of the company. This manuscript mainly selected the annual net cash flow from operating activities of listed companies as the main indicator affecting the company’s liquidity.

##### Total Debt (DEBT)

Total debt is a comprehensive indicator reflecting the company’s borrowing and is used to measure the company’s financial solvency. The company can exert the financial leverage effect through the debt and obtain more income using external paid capital. Therefore, the structure of debt size is reasonable and directly related to the company’s technological innovation.

##### Interest Coverage Ratio (INTE)

In a highly developed financial market, the company’s dependence on banks increases due to operating with debt. Suppose a company has no difficulty in paying interest on its debt. In that case, the company will raise funds more easily, and the cost of raising funds will be lower, so the company will be able to maintain a high debt ratio in its capital structure. In this manuscript, we have mainly used the interest coverage ratio to measure this, and a larger index indicates that the company’s solvency is strong and vice versa.

##### Asset Size (SIZE)

In academia, asset size is usually used to measure the size of a company. Similarly, this manuscript also applied it to measure the company’s size. Overall, the larger the size of the company’s asset size, the better the development and the higher the level of technological innovation. Given its large number, this manuscript treated the asset size as a logarithm in the empirical regression to eliminate the influence of the dimension.

##### A Number of Employees (LABOR)

The size of the labor force is an important factor affecting the technological innovation of a company. Overall, a large labor force is needed to achieve the scale effect in the early stage of a company’s development.

##### Age of the Company (AGE)

Listed companies are deeply influenced by years of establishment, and company’s age is considered to be an important variable affecting company technological innovation. This manuscript has expressed company’s age as the difference between the observation and company listing years.

### Quantity Sources and Descriptive Statistics

The panel data selected in this manuscript spanned from 2008 to 2017 and the companies listed in Shanghai and Shenzhen stock exchanges were selected through the database as the empirical samples. Following the common practice in related fields, the abnormal samples with missing important accounting data and the financial listed companies were excluded, and then the balanced panel data covering 6,270 observations were obtained for this manuscript. All data information was from the WIND database, CSMAR database, RESSET database, and annual financial reports of listed companies, and descriptive statistics of variables are shown in Table 1.

**TABLE 1:** Descriptive statistics.

Variables	Mean	Min	Max	Std. Dev.	N
INNO	17.2759	0.0000	25.3558	3.0416	6270
FUND	0.1212	0.0000	1.0000	0.3264	6270
GRANT	5.9688	−2.1203	11.9640	1.9981	6270
GROW	11.4113	−1,436.0220	99.3692	25.5567	6270
INCO	27.0969	−91.0644	8,269.9180	117.3292	6270
NET	20.3542	−19,511.3900	18,064.4300	566.5398	6270
DEBT	19.8943	0.0000	27.8210	1.7759	6270
INTE	1442.7490	−159,531.4000	1,844,918.0000	39224.3500	6270
SIZE	21.1182	0.0000	28.0699	1.5596	6270
LABOR	4.0382	2.5819	7.9460	0.9283	6270
AGE	3622.9230	26.0000	270,467.0000	16380.4700	6270

Wind Economic database pairs over 1.3 million macroeconomic and industry time series with powerful graphics and data analysis tools to give financial professionals the most comprehensive insights into China’s economy. CSMAR database is China Stock Market & Accounting Research Database. RESSET Database is a professional services data platform provider for model tests, investment research, etc. RESSET database was designed by several leading experts engaged in financial database and financial modeling research from Tsinghua University and Peking University. Overall, it has an empirical research-oriented design, with a design idea, system structure, data quality, and technical patterns reaching an international advanced level, and is conducted throughout full reference to the international renowned database but also involves the actual situation of China’s financial markets. As such, it can provide strong support for empirical, discipline, and laboratory research.

## Empirical Results and Analysis

Table 2 reports the econometric results of industrial investment funds, government R&D subsidies, and corporate technological innovation of listed enterprises in China from 2008 to 2017. Models (1–3) denote the regression results for the full sample, the sample with industrial fund involvement, and the sample without involvement, respectively. The following section provides an in-depth analysis of the regression results of each model separately.

**TABLE 2:** Empirical results.

INNOit	Model (1)	Model (2)	Model (3)

	Full samples	With industrial funds involvement	Without industrial funds involvement
FUNDit	0.1632**		
	(2.21)		
GRANTit	0.0293	0.1430***	0.0131
	(0.92)	(2.70)	(0.57)
GROWit	0.0008	0.0001	0.0009
	(0.01)	(0.02)	(1.00)
INCOit	0.0002**	−0.0001	0.0002
	(2.00)	(−0.06)	(1.00)
NETit	−0.0001	0.0001	−0.0001
	(1.00)	(0.50)	(1.00)
DEBTit	0.3310***	0.3960***	0.3230***
	(4.24)	(2.91)	(5.49)
INTEit	−0.2001***	0.0001	−0.0001
	(2.61)	(1.10)	(−1.41)
SIZEit	0.8490***	0.2400	0.9420***
	(6.24)	(1.21)	(11.00)
LABORit	0.2001***	0.0001	0.0001**
	(2.93)	(1.00)	(2.21)
AGEit	−0.0212	−0.0388	−0.0176
	(−1.19)	(−1.04)	(−1.11)
Intercept term	−7.6251***	2.4022	−9.1850***
	(−2.51)	(2.91)	(−2.48)
Individual effect	YES	YES	YES
Year Effect	YES	YES	YES
Coefficient of determination R2	0.36	0.39	0.36
F Value	40.93***	18.43***	123.57***
N	6270	760	5510

*,**, and ***Indicate significance at the 10, 5, and 1% levels, respectively; t-values are in parentheses below the estimated coefficients.

Firstly, the empirical results of industrial investment funds, government R&D subsidies, and firms’ technological innovations were analyzed in this manuscript. The results of model (1) show that the effect of industrial investment funds on the firm’s technological innovation is positive and significant at a 5% significance level, with a regression coefficient of 0.1632. This indicates that both industrial investment funds and government R&D subsidies help to improve a company’s technological innovation, confirming hypotheses 1 and 2.

Second, in terms of the presence and absence of industrial investment fund involvement, the results of model (2) reveal that the effect of government R&D subsidy on a firm’s technological innovation is positive, with the regression coefficient being 0.1430 at a 1% significance level. The results of model (3) show that the effect of government R&D subsidy on a firm’s technological innovation is positive, with a regression coefficient is 0.0131, but the effect is not significant. This indicates that government R&D subsidies of a company with industrial investment funds are conducive to promote its technological innovation level. At the same time, a company without industrial investment fund involvement also receives a positive effect on its technological innovation to some extent. Still, the regression results of companies without industrial investment fund involvement are not significant. Overall, the government R&D subsidies of listed companies in China mainly positively affect the technological innovation of listed companies, confirming hypothesis 3.

### Robustness Test

Table 3 reports the robustness test results of industrial investment funds, government R&D subsidies, and company technological innovation of listed companies in China from 2008 to 2017. Models (4–6) represent the regression results for the full sample, the sample with industrial fund involvement, and the sample without involvement, respectively. Since the econometric model contains first-order lagged terms of the explained variables, this manuscript adopted the GMM to solve the endogeneity problem of the model.

**TABLE 3:** Robustness test results.

INNOit	Model (4)	Model (5)	Model (6)

	Full sample	With industrial funds involvement	Without industrial funds involvement
INNOit-1	0.5705***	0.5588***	0.5500***
	(5.14)	(14.67)	(4.14)
FUNDit	0.1762**		
	(2.34)		
GRANTit	0.0130	0.0043	−0.0115
	(0.50)	(0.68)	(−1.03)
GROWit	0.0001	0.0027	0.0001
	(0.33)	(1.01)	(0.33)
INCOit	0.0001	0.0007	0.0001
	(1.00)	(0.10)	(1.00)
NETit	0.0001	0.0001	0.0001
	(1.00)	(1.00)	(1.00)
DEBTit	0.0647	0.1893*	0.0660
	(0.89)	(1.82)	(0.74)
INTEit	0.0001	−0.0001	0.0001
	(1.00)	(−1.00)	(1.00)
SIZEit	0.2933***	0.1942*	0.3026***
	(2.96)	(1.72)	(2.50)
LABORit	0.3001**	0.2201*	0.2301**
	(2.52)	(1.71)	(2.14)
AGEit	−0.0136	0.0090	−0.0137
	(−1.08)	(1.04)	(−1.11)
Intercept term	0.3784	0.2065	0.5064
	(1.12)	(1.26)	(1.50)
Individual effect	YES	YES	YES
Year Effect	YES	YES	YES
AR(1)	−2.6239***	−2.3642**	−2.2432***
	(0.0087)	(0.0312)	(0.0249)
AR(2)	0.80254	0.0702	0.7687
	(0.4222)	(0.4826)	(0.4421)
Sargan Test	26.0931	17.1954	25.9355
	(0.1202)	(0.3073)	(0.1136)
Wald value	2024.55***	759.18***	1844.10***
N	6270	760	5510

***, and ***Indicate significance at the 10, 5, and 1% levels, respectively; t-values are in parentheses below the estimated coefficients.

Specifically, this manuscript used the two-step systematic GMM estimation method to estimate the regression of the model with the tool variables as the first-order lagged terms of each explanatory variable and test the validity of the model with the tool variables using the Arellano-Bond (AR) (2) and Sargan values. The regression results of model (4) show that the AR(1) and AR(2) tests indicate the existence of first-order autocorrelation but not second-order autocorrelation for the resultant residual terms, where AR(1) is −2.6239 (*p* > chi^2^ = 0.0087) and AR(2) is 0.80254 (*p* > chi^2^ = 0.4222), so the GMM estimation is valid. Moreover, the Sargan test statistic for the tool variables is not significant at a 10% significance level, where the Sargan test is 26.0931 (*p* > chi^2^ = 0.1202), so the original hypothesis that the model is reasonable cannot be rejected, further indicating that the tool variables selected are valid. Similarly, models (5) and (6) could not reject the original hypothesis, so they were not repeated. Next, the results of the variables were analyzed in detail.

First, the empirical results of industrial investment funds, government R&D subsidies, and firms’ technological innovation were analyzed. The results of model (4) show that the effect of industrial investment funds on a firm’s technological innovation is positive and significant at a 5% level of significance, with a regression coefficient value of 0.1762. The effect of government R&D subsidies on a firm’s technological innovation is positive. The regression coefficient value of government R&D subsidies on a firm’s technological innovation is 0.0130, but it is not significant. The above results are more consistent with the previous findings and indicate that the findings of this manuscript are robust.

Secondly, the model (5) results show that the effect of government R&D subsidies on a firm’s technological innovation is positive, with a regression coefficient of 0.0043, but it is not significant. The results of model (6) indicate that the effect of government R&D subsidies on a firm’s technological innovation is negative, with a regression coefficient of −0.0115, but it is not significant. This indicates that government R&D subsidies of companies with industrial investment fund involvement are beneficial in promoting a high level of technological innovation within a company to some extent. On the contrary, companies without industrial investment fund involvement experience a negative effect in terms of technological innovation. However, the regression results of the companies without involvement are not significant. Therefore, government R&D subsidies of companies with industrial investment funds are conducive to the improvement of technological innovation, while companies without industrial investment funds experience an insignificant effect or even a “crowding-out effect” on technological innovation.

## Discussion

Finally, by synthesizing the empirical results of the above control variables, it is evident that, on the one hand, the effect of company growth on technological innovation is positive, indicating that there is a positive relationship between company growth ability and company technological innovation ability. The effect of sales revenue growth rate on a company’s technological innovation is positive, but the effect is very weak. Therefore, regardless of the involvement of industrial investment funds or not, the improvement of a company’s profitability helps to enhance its management and operation level to a certain extent and thus, improves a company’s technological innovation, which aligns with the hypotheses of this manuscript. The effect of net cash flow from operating activities on a company’s technological innovation is positive, but the estimated coefficient is very small, which means its effect is very limited. Therefore, increasing the net cash flow from operating activities is not an effective way to enhance a company’s technological innovation, which also shows that the enhancement of a company’s technological innovation essentially requires its own involvement, rather than its own capital liquidity. The positive effect of total debt on a company’s technological innovation indicates that increasing a company’s total debt helps to promote its technological innovation level. The interest coverage ratio has a positive effect on technological innovation, but the effect is very limited. In addition, expanding a company’s size and increasing the number of employees are conducive to improve its management and operation level and enhance its technological innovation. This also suggests that the current companies with industrial fund involvement in China have stronger innovation ability and sufficient innovation motivation than those that do not, which indicates the former companies’ technological innovation receives a significant promotion effect from another aspect beyond their funding source.

On the other hand, increasing the number of listing years does not necessarily promote technological innovation. This is because, with the development of the listed companies, the number of patented technologies also increases. Therefore, companies are less motivated to engage in technological innovation activities, making them less motivated to innovate.

## Conclusion

Industrial investment funds are a new financing innovation model that can build an effective financing channel for enterprises. This manuscript has empirically analyzed the effect of industrial investment fund involvement and government R&D subsidies on company technological innovation by using the panel data of listed companies in China from 2008 to 2017. The study shows that, first, the involvement of industrial investment funds can increase a company’s R&D investment by providing financial capital for the company, which can effectively reduce the shortage of funds in the process of technological innovation and ensure the normal development of innovation activities. Second, government R&D subsidies can alleviate the pressure of a company’s R&D investment to a certain extent, which can help to promote its technological innovation level. Third, for companies with industrial investment funds, government R&D subsidies can assist to promote technological innovation to a certain extent. In contrast, for companies without an industrial investment fund, government R&D subsidies do not significantly affect technological innovation or even have a “crowding-out effect.”

### Policy Recommendations

This manuscript proposed the following policy recommendations based on the research findings. First, policymakers should clarify the orientation of industrial investment funds, explore a more suitable industrial investment funds model for China’s market, broaden the financing channels of industrial investment funds, enhance the financing capacity of industrial investment funds management companies, maximize the positive effect of industrial investment funds in the process of company technological innovation, and guide companies to increase their R&D investment. Secondly, policy decisions should strive to improve the accuracy of government R&D subsidies, make full use of the movement of industrial investment funds to enhance the efficiency of government R&D subsidies, and improve the technological level of Chinese listed companies. Third, the relevant authorities must regulate the operation system of industrial investment funds, which is a policy-based financial tool, further improves the regulatory system of industrial investment funds in China, and fully guarantees the balance between the interests of all parties of industrial investment funds.

## Data Availability Statement

The original contributions presented in this study are included in the article/supplementary material, further inquiries can be directed to the corresponding author.

## Author Contributions

The author confirms being the sole contributor of this work and has approved it for publication.

## Conflict of Interest

The author declares that the research was conducted in the absence of any commercial or financial relationships that could be construed as a potential conflict of interest.

## Publisher’s Note

All claims expressed in this article are solely those of the authors and do not necessarily represent those of their affiliated organizations, or those of the publisher, the editors and the reviewers. Any product that may be evaluated in this article, or claim that may be made by its manufacturer, is not guaranteed or endorsed by the publisher.

## Footnotes

1 As defined by the National Development and Reform Commission (2006), an industrial investment fund is a financial investment system that invests in the shares of unlisted companies and provides related financial management services, thereby achieving shared risk and gaining returns, usually by establishing a fund company and issuing the relevant fund shares to the general public investors after which the fund company chooses to act as the manager of the fund itself, or separately entrusts fund manager to effectively manage the underlying assets of the fund.

2 At present, government incentives are mainly in the form of government R&D subsidies, financial subsidies, R&D subsidies, policy subsidies, and tax deductions, etc., among which government R&D subsidies as the “supporting hand” of listed companies have been a key topic of research by scholars in China and internationally.
